# Letter to Editor From Ishaq MU et al: “Semaglutide Concurrently Improves Vascular and Liver Indices in Patients With Type 2 Diabetes and Fatty Liver Disease”

**DOI:** 10.1210/jendso/bvae217

**Published:** 2024-12-13

**Authors:** Muhammad Umer Ishaq, Noor Fatima, Rida Sharif, Warda Awais

**Affiliations:** Nishtar Medical College, Nishtar Medical University, Punjab 66000, Pakistan; Nishtar Medical College, Nishtar Medical University, Punjab 66000, Pakistan; Nishtar Medical College, Nishtar Medical University, Punjab 66000, Pakistan; Nishtar Medical College, Nishtar Medical University, Punjab 66000, Pakistan

We meticulously scrutinized the work by Korakas et al [1] that investigated semaglutide's benefits in improving both vascular and liver indices in individuals with type 2 diabetes (T2D) and metabolic dysfunction associated steatotic liver disease (MASLD). We commend the authors for their pioneering work investigating semaglutide's effects in humans with a 12-month follow-up period. We would like to bring attention to a few methodological limitations that may guide the readers to interpret the study findings such as the small sample size of only 75 with 25 patients receiving dipeptidyl peptidase-4 inhibitor and 50 undergoing 1-mg semaglutide treatment, which might have reduced the statistical power to detect any significant change in liver stiffness. Also, the treatment group (50) was twice the size of the control group (25) with no true placebo usage, which might have led to biased estimation and limited statistical power. Moreover, they did not employ liver biopsy, which is a gold standard for assessing liver fibrosis and steatosis; FibroScan is noninvasive but may not provide accurate staging. They could have used indices like the AST-Platelet ratio index, hepatic steatosis index, and fatty liver index along-with fibrosis-4 index (FIB-4) for MASLD evaluation, which might have improved accuracy in detecting and staging liver disease. They could have considered confounding variables like diet, physical activity, and liver pathology history, as these might have affected the results [1].

Similar to the Newsome study [2] (59% nonalcoholic steatohepatitis resolution) with semaglutide doses (0.1-0.4 mg), this study demonstrated semaglutide's benefit on liver health (improved controlled attenuation parameter and FIB-4 scores).While the Newsome study reported improvement in weight loss (13% mean weight loss) and glycemic control, this study also showed vascular indices betterment.

Glucagon-like peptide-1, an intestinal hormone, regulates blood sugar levels by stimulating insulin release, improving insulin function, enhancing glucose tolerance, and reducing glucagon release [3]. A recent review by Almutairi et al [4] elucidated that glucagon-like peptide 1 receptor (GLP-1R) agonists reduce vessel proliferation, decrease oxidative stress, and increase nitric oxide production, contributing to their antihypertensive and antiatherosclerotic effects. These effects were validated in the SUSTAIN-6 109-week clinical trial, in which semaglutide was revealed to curb the risk of cardiovascular mortality [5].

GLP-1R agonists exhibit hepatoprotective effects by mitigating lipotoxicity in the liver and improve glucagon resistance by enhancing β-oxidation and reducing lipogenesis, inducing depressed hepatic triacylglycerol levels, offering a potential therapeutic strategy for managing MASLD [6]. In a clinical trial spanning 72 weeks by Ratziu et al [7], semiglutide's 0.4-mg treatment effects unraveled steatohepatitis resolution, observed by pathologists along with machine learning–derived categorical assessments, and fibrosis reduction. Another study by Engström et al [8] showed that treatment with GLP-1R agonists was linked to a significant decrease in the incidence of both compensated and decompensated cirrhosis.

Based on these results, more monotherapy or combination trials involving diverse populations on a large scale are necessary to elucidate the protective effects of semaglutide, further navigating the treatment therapy for people with T2D, and the potential of this drug to safeguard from the complications of T2D.

## Disclosures

No disclosures.

## Abbreviations

FIB-4: fibrosis-4 index
GLP-1R: glucagon-like peptide 1 receptor
MASLD: metabolic dysfunction associated steatotic liver disease
T2D: type 2 diabetes

## References

[1] Korakas E, Kountouri A, Pavlidis G, et al Semaglutide concurrently improves vascular and liver indices in patients with type 2 diabetes and fatty liver disease. J Endocr Soc. 2024;8(8):bvae122. 10.1210/jendso/bvae122 PMC11228545 38979402

[2] Newsome PN, Buchholtz K, Cusi K, et al A placebo-controlled trial of subcutaneous semaglutide in nonalcoholic steatohepatitis. N Engl J Med. 2021;384(12):1113‐1124. 33185364 10.1056/NEJMoa2028395

[3] Mahapatra MK, Karuppasamy M, Sahoo BM. Therapeutic potential of semaglutide, a newer GLP-1 receptor agonist, in abating obesity, non-alcoholic steatohepatitis and neurodegenerative diseases: a narrative review. Pharm Res. 2022;39(6):1233‐1248. 35650449 10.1007/s11095-022-03302-1 PMC9159769

[4] Almutairi M, Al Batran R, Ussher JR. Glucagon-like peptide-1 receptor action in the vasculature. Peptides. 2019;111:26‐32. 30227157 10.1016/j.peptides.2018.09.002

[5] Marso SP, Bain SC, Consoli A, et al Semaglutide and cardiovascular outcomes in patients with type 2 diabetes. N Engl J Med. 2016;375(19):1834‐1844. 27633186 10.1056/NEJMoa1607141

[6] Lin YH, Zhang ZJ, Zhong JQ, et al Semaglutide combined with empagliflozin vs. monotherapy for non-alcoholic fatty liver disease in type 2 diabetes: study protocol for a randomized clinical trial. PLoS One. 2024;19(5):e0302155. 38701096 10.1371/journal.pone.0302155 PMC11068176

[7] Ratziu V, Francque S, Behling CA, et al Artificial intelligence scoring of liver biopsies in a phase II trial of semaglutide in nonalcoholic steatohepatitis. Hepatology. 2024;80(1):173‐185. 38112484 10.1097/HEP.0000000000000723 PMC11185915

[8] Engström A, Wintzell V, Melbye M, et al Association of glucagon-like peptide-1 receptor agonists with serious liver events among patients with type 2 diabetes: a Scandinavian cohort study. Hepatology. 2024;79(6):1401‐1411. 38085855 10.1097/HEP.0000000000000712

